# Urine Sodium Excretion in Children with Primary Hypertension: A Single-Center Retrospective Study

**DOI:** 10.3390/jcm14248643

**Published:** 2025-12-05

**Authors:** Marcin Sota, Marta Armuła, Michał Szyszka, Piotr Skrzypczyk

**Affiliations:** 1 Student Scientific Group, Department of Pediatrics and Nephrology, Medical University of Warsaw, 02-091 Warsaw, Poland; s087180@student.wum.edu.pl (M.S.); s082343@student.wum.edu.pl (M.A.); 2 Department of Pediatrics and Nephrology, Doctoral School, Medical University of Warsaw, 02-091 Warsaw, Poland; 3 Department of Pediatrics and Nephrology, Medical University of Warsaw, 02-091 Warsaw, Poland; pskrzypczyk@wum.edu.pl

**Keywords:** primary hypertension, peadiatric hypertension, urinary sodium excretion, adolescence, high blood pressure, ambulatory blood pressure monitoring, uric acid, antihypertensive medications

## Abstract

**Background**: Pediatric hypertension is an increasingly recognized health concern and is commonly influenced by modifiable factors such as dietary sodium intake and obesity and non-modifiable factors like family history of hypertension. Urinary sodium excretion provides an objective surrogate marker of sodium consumption and may be associated with blood pressure severity. This study aimed to evaluate urinary sodium excretion in children with primary hypertension (PH) and to test the hypothesis that higher sodium excretion is associated with less favorable clinical, biochemical, and blood pressure parameters. **Methods**: This retrospective, cross-sectional, single-center study analyzed data from 369 hypertensive patients and 59 healthy children. Patients with a confirmed diagnosis of PH and ambulatory blood pressure monitoring results were included in the study group. Clinical, anthropometric, laboratory, echocardiographic, and blood pressure data were examined, and sodium excretion was evaluated using both the spot urine sodium-to-creatinine ratio and 24-h urinary sodium per kilogram of body weight. **Results**: Children with hypertension exhibited higher urinary sodium excretion compared to the control group. Sodium excretion of the hypertensive group, measured using the sodium/creatinine ratio and 24 h urinary sodium excretion per kilogram, was positively correlated with 25-hydroxyvitamin D, the urinary potassium/creatinine ratio, and the urinary uric acid/creatinine ratio. Moreover, negative correlations were observed for both parameters with age, body weight, serum uric acid, and left ventricular mass. In the multivariate analysis, weighted Z-score (beta = −0.393), age (beta = −0.293), 25-OHD (beta = 0.182), and arterial hypertension in the father (beta = 0.166) predicted 24 h urinary sodium excretion. Children with excessive sodium excretion had a significantly higher systolic blood pressure load over 24 h. **Conclusions**: Urinary sodium excretion is elevated in children with PH. Children with a lower weight for their age, who are younger, and who have a father with arterial hypertension might be at higher risk of excessive urine excretion. Our findings underscore the clinical importance of dietary sodium reduction as a non-pharmacological therapeutic target, especially in these patient populations. Prospective studies are needed to evaluate its impact on long-term cardiovascular outcomes in this population.

## 1. Introduction

Arterial hypertension (AH) is being increasingly recognized not only as a condition affecting adults but also as an increasing health issue in children and adolescents. Hypertension in children is operationally defined as systolic or diastolic blood pressure equal to or exceeding the 95th percentile, adjusted for height, age, and sex according to normative reference standards [1]. Pediatric hypertension differs from hypertension in adults in that it is not characterized by a specific cutoff value of ≥140/90 mmHg; rather, it is defined by the child’s age, height, and sex [2].

Recent studies report an overall global prevalence of roughly 4% for hypertension among the pediatric population [2]. Moreover, a rise in the prevalence of pediatric hypertension has been observed over the last three decades, primarily as a result of contemporary lifestyles [2]. Hypertension in children and adolescents is now recognized as a significant global health concern due to the increase in childhood obesity and lack of physical activity [3]. Additionally, obese children have a significantly higher risk of developing hypertension compared to non-obese children [4]. Furthermore, untreated hypertension in children increases the risk of developing adult hypertension and other chronic diseases later in life [5].

The renin–angiotensin–aldosterone system (RAAS) is the primary regulator of blood pressure and sodium levels in a state of physiological homeostasis. High sodium intake increases intravascular volume and water retention. This raises cardiac output and, consequently, inhibits RAAS to some extent. This facilitates sodium excretion, among other means, through urine [6]. Excess dietary sodium is a well-documented factor leading to the development of hypertension [1]. Additionally, it was shown to stimulate the immune system and the sympathetic nervous system and impair renal sodium excretion [7,8]. Moreover, in both children and adults, a congenital defect that results in a reduction in nephron number or direct kidney damage can reduce the efficacy of natriuresis, making the affected individual sensitive to excess sodium load [9]. In such cases, reduced natriuresis leads to higher body fluid volume and blood pressure, which causes further damage to the kidneys, trapping the patient in a vicious cycle.

The current clinical evidence suggests that lowering sodium intake in children and adolescents results in a small but significant immediate reduction in blood pressure, which in turn reduces the risk of developing cardiovascular diseases in adulthood [10]. A meta-analysis conducted by Leyvraz et al. found that for every additional gram a child consumes in their daily diet, their systolic blood pressure rises by 0.9 mmHg and diastolic blood pressure by 0.7 mmHg [11]. Additionally, another meta-analysis concluded that for children with elevated blood pressure (BP) without an identifiable cause, an additional 1 g of sodium per day causes an elevation in both systolic and diastolic blood pressure, highlighting the increased sensitivity to dietary sodium in this patient group [12]. Numerous studies have suggested that restricting dietary sodium consumption can result in a small but significant decrease in blood pressure in children across various age groups, including infants, primary school children, and adolescents [13,14,15]. Furthermore, epidemiological studies involving tens of thousands of adult patients have demonstrated an association between increased urinary sodium excretion and higher blood pressure [6].

Therefore, this study aimed to evaluate sodium urinary excretion in pediatric patients with primary hypertension under the care of a tertiary nephrology center and test the hypothesis that higher sodium excretion is associated with less favorable clinical, biochemical, and blood pressure parameters.

## 2. Materials and Methods

### 2.1. Study Design

**Figure 1:**
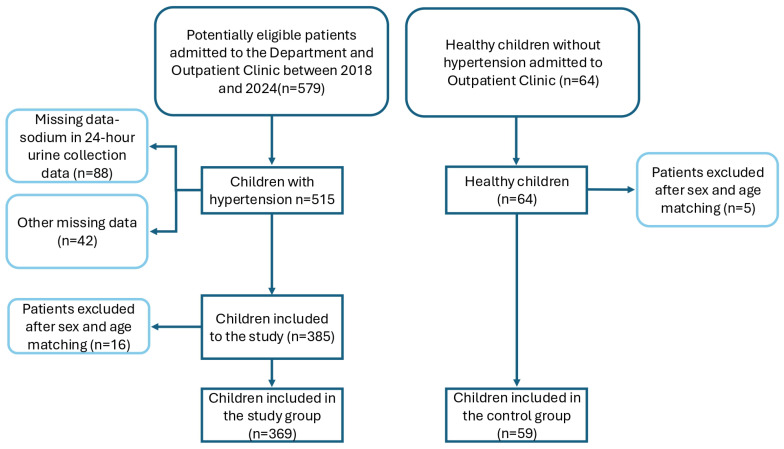
Participants’ flow diagram.

This retrospective, cross-sectional, single-center study included data from children and adolescents diagnosed with arterial hypertension (AH), alongside a control group of healthy individuals. The number of participants included in this study are presented in the flow diagram (Figure 1). Of the 515 potentially eligible patients, 369 were enrolled in the final analysis. The control group consisted of 59 healthy age- and gender-matched patients.

**Figure 2:**
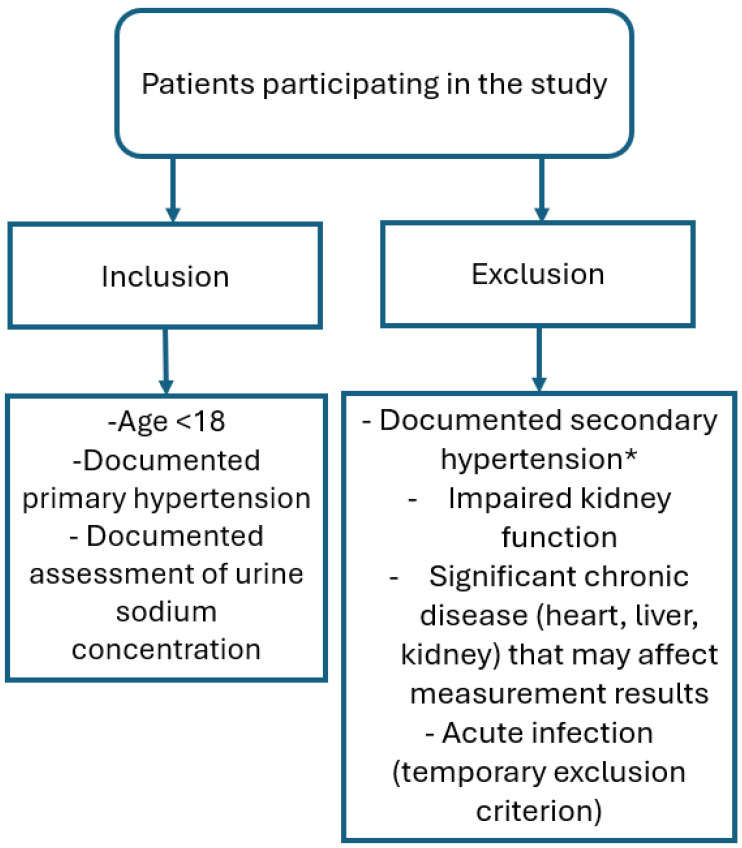
The inclusion and exclusion criteria of patients initially enrolled in the study. Secondary forms of hypertension were diagnosed based on [1,16]. * The following exams were performed: abdominal ultrasound with kidney and artery Doppler imaging, ions, gases, creatinine, cystatin C, urea, metanephrines, renin, aldosterone, echocardiography with assessment of the aortic arch, urine steroid profile, thyroid hormones, and vitamin D3 metabolites.

Medical records of all patients referred to the tertiary Department of Nephrology between 2018 and 2024 with a confirmed diagnosis of hypertension, as defined by the European Society of Hypertension (ESH) guidelines [1] and verified by ambulatory blood pressure monitoring (ABPM), were reviewed. Eligibility criteria for inclusion in the study included both a confirmed diagnosis of hypertension and a documented assessment of urinary sodium excretion. Patients with secondary forms of hypertension, impaired kidney function, significant chronic disease (the heart, liver, or kidney) that may affect the measurement results, or acute infections (temporary exclusion criterion), as well as those whose urine sodium was not measured or whose documentation lacked critical information (e.g., ABPM, recorded blood pressure values, blood tests), were excluded from the study. For this analysis, only data obtained during the patients’ initial hospital admission were considered. The inclusion and exclusion criteria are depicted in Figure 2.

### 2.2. Data Collection

The collected clinical data included sex, age (in years), duration of hypertension (in months), height (in cm), weight (in kg), and body mass index (BMI, in kg/m^2^). Anthropometric measures were standardized using Polish reference data and expressed as Z-scores [17]. Overweight and obesity were classified based on the World Health Organization BMI Z-score thresholds of >1 and >2, respectively [18]. Information regarding the use of antihypertensive medication at the time of examination was also recorded. Due to the retrospective nature of the study, a reliable analysis of diet and dietary sodium intake was not accessible.

The laboratory parameters analyzed in serum comprised sodium (mmol/L), potassium (mmol/L), calcium (mg/dL), 25-hydroxyvitamin D [pg/mL], creatinine (mg/dL), uric acid (mg/dL), total cholesterol (mg/dL), both HDL- and LDL-cholesterol (mg/dL), and triglycerides (mg/dL). Vitamin D was assessed by chemiluminescence (ARCHITECT i1000SR, Abbott Park, IL, USA). Normative pediatric values for lipid metabolism were applied [19].

Urinary analyses included measurements of sodium, potassium, calcium, phosphorus, uric acid, creatinine, and albumin. These results were expressed as ratios relative to creatinine within the same sample, including Na/crea (mmol/mg), K/crea (mmol/mg), Ca/crea (mg/mg), P/crea mg/mg), and UA/crea (mg/mg), alongside the albumin-to-creatinine ratio (ACR, mg/g). Pathological albuminuria was defined as an ACR exceeding 30 mg/g [1]. Sodium urinary excretion was calculated from the Na/Crea (mmol/mg) ratio and from daily urinary collection (mmol/kg/24 h). The cutoff value for sodium urine excretion per 24 h per kilogram was adapted as >4 mmol/kg/24 h [20].

Peripheral blood pressure was assessed using an oscillometric device: Omron HBP-1320 (OMRON Healthcare, Kyoto, Japan). Measurements were expressed in mmHg and compared to age-, sex-, and height-specific normative values [17], subsequently converted to Z-scores.

Ambulatory blood pressure monitoring over 24 h was performed using the SUNTECH OSCAR 2 device (SunTech Medical, Inc., Morrisville, NC, USA), with interpretation based on the American Heart Association (AHA) recommendations [21]. Measurements were taken every 15 min between 6:00 AM and 10:00 PM and every 30 min from 10:00 PM to 6:00 AM. Sleep and wake periods were determined individually through patient diaries. Evaluated ABPM parameters included 24 h systolic, diastolic, and mean arterial pressure (SBP, DBP, MAP) in mmHg and Z-scores [22], blood pressure load over 24 h (%), nocturnal blood pressure dip (%), and blood pressure variability. An abnormal BP load was considered a value exceeding 25% of readings above the 95th percentile for height [21]. A nocturnal BP dip was calculated as the percentage reduction in average systolic and diastolic pressures during sleep compared to daytime values. A dip of less than 10% was classified as a disturbed circadian blood pressure rhythm [1].

Echocardiographic examinations were performed using a Philips iE33 xMATRIX system (Philips Healthcare, Amsterdam, The Netherlands) with transducers adjusted to the patient’s body size, following the same standardized protocol used in the hospital for both the study and control groups. The left ventricular mass index (LVMI) was calculated and indexed to height^2.7^ for participants aged ≤15 years and to body surface area (BSA) for those ≥ 16 years, with BSA calculated using the DuBois and DuBois formula. Left ventricular hypertrophy (LVH) was defined as an LVMI exceeding the 95th percentile for age and sex in children or >115 g/m^2^ in boys and >95 g/m^2^ in girls aged 16 years or older [23].

The study was conducted as an extension of a previously approved research project (Ethics Committee approval No. KB/58/2016) and adhered to the principles outlined in the Declaration of Helsinki. Written informed consent was obtained from all participants aged ≥16 years and from legal guardians for younger children, with consent forms signed upon hospital admission.

### 2.3. Statistical Analysis

All statistical procedures were conducted using Dell Statistica 13.0 PL (Dell Inc., Aliso Viejo, CA, USA). Continuous variables were described as means with standard deviations (SD) and interquartile ranges (IQR). The Shapiro–Wilk test was employed to assess the normality of the distribution. Between-group comparisons for normally distributed variables were performed using Student’s *t*-test, while non-normally distributed data were analyzed using the Mann–Whitney U test. Pearson’s correlation coefficient was used to assess relationships between parametric variables, and Spearman’s rank correlation was used for non-parametric data. Multivariate analysis of factors related to sodium urinary excretion was performed using logistic regression and a generalized regression model (GRM). A *p*-value of less than 0.05 was considered to indicate statistical significance.

### 2.4. Ethical Considerations

The data used in this study were collected as part of an ongoing project approved by the institutional review board in March 2016, which is still in progress. For the purposes of this article, we analyzed the data retrospectively, selecting patients who had their urinary sodium levels measured. According to the regulations of our university’s bioethics committee, additional approval is not required for retrospective analyses of previously collected data. Therefore, this retrospective study was submitted for notification to the Ethics Committee of the Medical University of Warsaw only.

## 3. Results

### 3.1. Clinical Characteristics of the Study Group

The clinical characteristics of the study group are presented in Table 1. Patients with hypertension and patients in the control group did not differ in terms of sex, age, and height. Hypertension was more prevalent in males (66.8% of patients with hypertension were male and 33.2% female). Being overweight was found in 26.3% of patients (99 with hypertension and 18 in patients in the control group), and obesity was found in 16.4% patients (60 with hypertension and 13 in patients in the control group). The study group had significantly higher weight, BMI, and respective Z-scores compared to the control group. HDL cholesterol was higher in the control group, while serum sodium and serum uric acid were higher in the study group. Total LDL, cholesterol, calcium, and creatinine levels did not differ between groups. A total of 95 patients had decreased HDL cholesterol levels, and elevated total cholesterol was found in 88 children, elevated LDL cholesterol in 42 children, and elevated triglyceride levels in 133 children. The study group had a higher serum sodium-to-creatinine ratio, potassium-to-creatinine ratio, and phosphorus-to-creatinine ratio, with no differences in the calcium-to-creatinine ratio and urine acid-to-creatinine ratio. Both blood pressure values, outpatient checkups, and most of the 24 h ambulatory measurements were consistently elevated in the study group. However, the 240 h heart rate and nocturnal systolic and diastolic blood pressure dips showed no significant differences between the groups. Pulse pressure and left ventricular mass z-score were notably higher in the hypertensive group.

**Figure 3:**
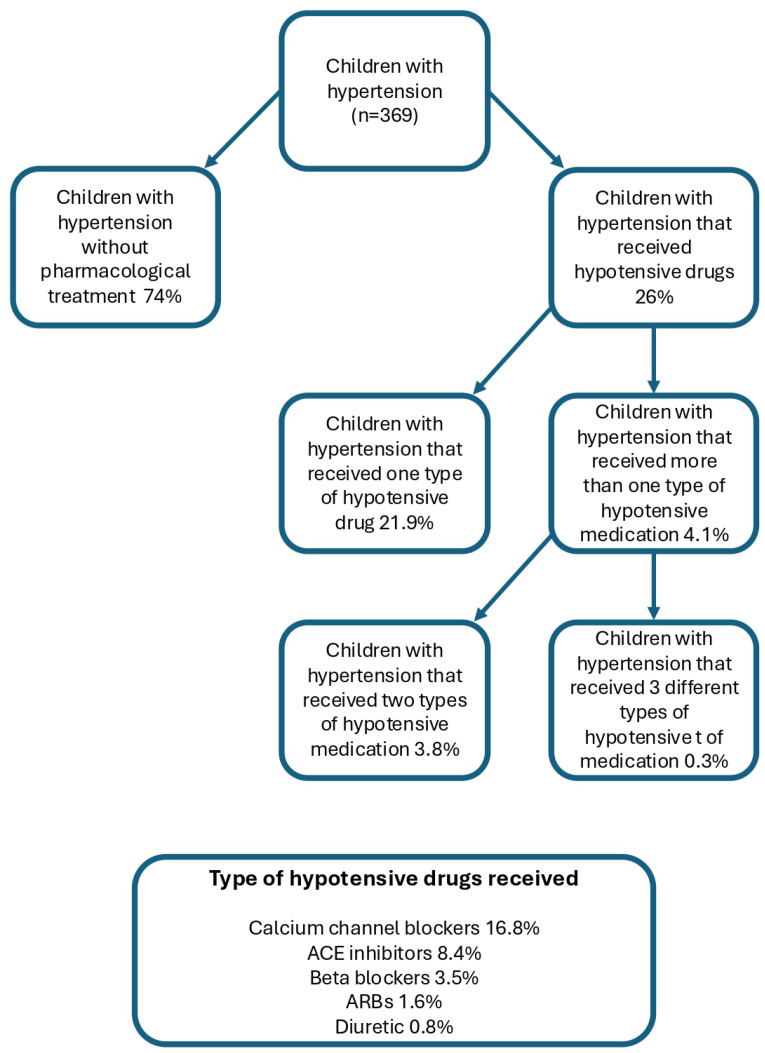
Distribution of antihypertensive medication use.

Of all patients included in the study group, 26.0% (96 out of 369) received at least one hypotensive drug or a combination of hypotensive drugs. Furthermore, 16.8% patients received calcium channel blockers (62 out of 369), 8.4% received ACE inhibitors (31 out of 369), 3.5% received beta blockers (13 out of 369), 1.6% received ARBs (6 out of 369), and 0.8% received a diuretic drug (3 out of 386). In addition, 4.1% (15 out of 369) of patients received more than one type of hypotensive medication, and 0.3% (1 out of 369) of patients received three different types of hypotensive medicines. A graphical summary of antihypertensive medication use in the study group is presented in Figure 3.

### 3.2. Correlation of Sodium to Creatinine Urine Excretion

The correlations of sodium urinary excretion for the study group are presented in Table 2 and Table 3. Firstly, the urinary sodium-to-creatinine ratio (Na/Creat) was significantly correlated with 24 h urinary sodium excretion, expressed in mmol/kg/24 h (r = 0.420, *p* < 0.001). Several parameters correlated positively with both the sodium/creatinine ratio and the 24 h urinary sodium excretion per kilogram of body weight, such as 25-hydroxycholecalciferol (25 OHD), the urinary potassium/creatinine ratio (K/Creat), and the urinary uric acid/creatinine ratio (UA/Creat). Positive correlations were observed only with the sodium/creatinine index for SBP Z-score, duration of symptoms, ACE inhibitor use, Z-score LVM, urinary calcium/creatinine ratio (Ca/Creat), and albumin/creatinine ratio (ACR). Parameters such as cholesterol, HDL cholesterol, urine volume, and urinary phosphorus/creatinine ratio (P/Creat) correlated positively with 24 h sodium excretion per kilogram. Negative correlations were found for both sodium/creatinine and 24 h urinary sodium excretion per kilogram of body weight for age, serum uric acid, left ventricular mass (LVM), and body weight. Parameters that correlated negatively only with the sodium/creatinine index included height, serum and urinary creatinine, serum potassium, and mean arterial pressure (MAP). BMI, diastolic blood pressure (DBP), DBP Z-score, parental hypertension, antihypertensive drug use, calcium channel blocker use, and triglycerides showed negative associations exclusively with 24 h sodium excretion per kilogram.

In the multivariate analysis, 24 h urinary sodium excretion was predicted significantly by weighted Z-score (beta = −0.393, 95% confidence interval—95CI: −0.562–−0.224), age (beta = −0.293, 95CI: −0.450–−0.135), 25 OHD (beta = 0.182, 95CI: 0.042–0.322), and the presence of arterial hypertension in the father (beta = 0.166, 95CI: 0.027–0.305).

### 3.3. Excessive Sodium Excretion Group

A subgroup of 19 patients with excessive urinary sodium excretion (24 h urinary sodium > 4 mmol/kg/24 h) was identified and compared to those with normal sodium excretion, as presented in Table 4. Patients with excessive sodium excretion were significantly younger, shorter, and had lower BMI values. Among pressure-related parameters, only the 24 h systolic blood pressure load (SBPL/24 h) was significantly higher in the excessive sodium group; all other systolic and diastolic pressure values did not differ between groups. Triglyceride, plasma potassium, and HDL cholesterol levels were also significantly higher in the excessive sodium group. Although not statistically significant, patients with excessive sodium excretion showed a trend toward higher 24 h heart rates and greater systolic and diastolic blood pressure dips. In the multivariate analysis, age (odds ratio—OR: 0.666, 95CI: 0.556–0.797) and triglyceride concentration (OR: 0.974, 95CI: 0.955–0.993) were the only significant predictors of excessive urinary sodium excretion.

### 3.4. Age-Stratified Analysis

We performed a separate analysis comparing younger and older children—above and below 12 years of age. A separate comparative analysis was performed between these two groups. The results are presented in Table 5. The groups differed in terms of gender distribution. In the younger group, girls and boys were affected by hypertension equally as often. In the older group, however, a significant proportion of patients were boys. Younger patients, i.e., those under 12 years of age, were also relatively taller than older patients (Z-score comparison). Adolescents presented with higher uric acid levels. Despite their younger age, younger patients had higher total and LDL cholesterol. Compared to older children, younger children excreted greater amounts of sodium in their urine. The groups did not differ in terms of the number of children who required pharmacological treatment. However, ACE inhibitors were a significantly more common drug in the younger group.

## 4. Discussion

Our single-center cross-sectional analysis evaluated serum and urinary sodium in a large group of pediatric patients with primary hypertension. The primary finding of the study indicated that both sodium concentration and urinary sodium excretion were significantly elevated in children with hypertension in comparison to the healthy controls. We compared the amount of sodium excreted in children with hypertension, while simultaneously analyzing the correlation between the urinary sodium-to-creatinine ratio in a spot urine sample and total sodium excretion per kilogram of body weight over a 24 h period. We found numerous correlations between sodium excretion and body weight, blood biochemistry, and left ventricular mass. Contrary to intuition, we demonstrated numerous negative correlations between urinary sodium excretion and blood pressure, as well as left ventricular mass. In the multivariate analysis, 24 h urinary sodium excretion was predicted significantly by weight, age, vitamin D supply, and the presence of arterial hypertension in the father. Only patients with excessive sodium excretion had elevated systolic blood pressure loads over 24 h compared to patients with normal sodium excretion.

In our study, we assessed sodium excretion in urine using both 24 h urine collection and the sodium-to-creatinine ratio. It is not possible to perform a 24 h urine collection in all patients, e.g., in patients with an intellectual disability, young children, or children who wet themselves. On the other hand, the sodium-to-creatinine ratio can be performed in every child. There is considerable doubt in the literature as to whether the sodium-to-creatinine ratio can replace daily urine collection. Some authors have demonstrated a high correlation between spot urine samples and daily urine collections [24,25], while others have not [26]. Since sodium excretion in urine is not constant, it seems that increasing the number of measurements, e.g., two 24 h urine collections, may provide a more accurate reflection of sodium intake. Therefore, in our study, we analyzed only those patients who had both daily urine collection and sodium-to-creatinine ratio available from urine samples. It is worth noting that in our cohort of patients with PH we found a positive correlation between these two indicators of sodium urinary loss.

There is no consensus on whether a single measurement of sodium in urine (spot or daily urinary collection) is a good estimator of both sodium intake and cardiovascular risk. Large population studies in adults have shown a weak positive correlation (mainly systolic pressure) with urinary sodium excretion [27,28]. In a study published in 2022 involving more than 10,000 adults who had at least two daily urine collections assessed, high sodium excretion and low potassium excretion were associated in a dose–response manner with a higher cardiovascular risk [29]. Similar conclusions can be drawn from a meta-analysis published two years earlier [30]. On the other hand, there is some evidence that low urinary sodium excretion is associated with a higher cardiovascular risk. This inverse relationship may be a consequence of excessive activation of the RAA system in patients with dietary sodium deficiency [31].

Similarly, no clear correlation has been demonstrated between blood pressure and natriuresis in children. For example, the work of Polish authors has shown a negative correlation between blood pressure and urinary sodium excretion in children with type 1 diabetes [32]. Although a study conducted 45 years ago showed a positive correlation between sodium in urine and blood pressure in children, the authors analyzed as many as seven daily urine samples from each subject [33]. Similarly, in our study, it is challenging to establish a clear direction of dependence between natriuresis, blood pressure, and organ changes. Further prospective studies are necessary. It is undoubtedly essential to assess sodium excretion at multiple time points (two or even seven) in conjunction with simultaneous dietary sodium evaluation.

We observed a negative correlation between body weight and BMI, as well as between 24 h urinary sodium excretion and body weight. This finding may appear counterintuitive, as children with higher BMI are often expected to consume more sodium [34]. The observed inverse correlation may be due to the mathematical effect of expressing sodium excretion relative to body weight.

Some of the patients included in our study had already initiated pharmacological treatment for arterial hypertension before the assessment of biochemical and clinical parameters. This could have influenced the results, particularly regarding urinary sodium excretion and blood pressure measurements, especially in cases where medications known to affect sodium handling were used [35]. Our study found higher urinary sodium concentrations in children with hypertension than in their normotensive peers. In a study conducted among healthy Spanish children, no significant association was found between 24 h urinary sodium excretion and blood pressure [36]. Nevertheless, the study did not include children with diagnosed hypertension, which may limit the generalizability of the findings to high-risk populations. In contrast, more recent studies focusing specifically on hypertensive children have shown a significant relationship between sodium excretion and blood pressure level. A study published in 2022 reported higher 24 h urinary sodium excretion in children with primary hypertension compared to their normotensive peers with systolic blood pressure [37].

Our study revealed a negative correlation between age and the urinary sodium-to-creatinine ratio (Na/creat), indicating that older children excrete less sodium relative to creatinine. This finding is consistent with previous scientific studies [38].

A notable finding of our study is the significant negative correlation between serum uric acid levels and both the urinary sodium-to-creatinine ratio and 24 h urinary sodium excretion per kilogram of body weight. This suggests that higher serum uric acid levels are associated with lower urinary sodium excretion. It is suspected that high serum uric acid levels are associated with high blood pressure, especially in patients with primary hypertension. [39]. However, the aforementioned study was conducted in a cohort comprising children with primary hypertension, secondary hypertension, and white coat hypertension. A strong correlation between serum uric acid levels and blood pressure was observed in the vast majority (90%) of children with primary hypertension. In contrast, only about 30% of children with secondary hypertension exhibited such a correlation. Moreover, a key methodological difference was that the patients included had not yet initiated antihypertensive treatment. The absence of pharmacological influence allowed for an assessment of sodium excretion and metabolic parameters under baseline conditions, providing a valuable reference point when interpreting our findings, especially considering the potential effects of medication on sodium retention and biochemical profiles [35].

Another interesting observation is that patients receiving antihypertensive therapy exhibited normal urinary sodium levels; none were among the 19 individuals with elevated sodium concentrations. This phenomenon may be attributed to the fact that patients on long-term antihypertensive therapy are more likely to adhere to a low-sodium diet and follow medical recommendations regarding lifestyle modification. Sodium intake is closely correlated with sodium excretion, a relationship that has been confirmed in controlled settings and a strictly controlled environment [40,41]. However, hypertensive and non-hypertensive patients tend to underreport sodium consumption; therefore, a questionnaire-based evaluation could result in inaccurate results [42,43].

In our study, 26.0% of patients with primary hypertension were treated with at least one type of hypotensive medication; this result is slightly lower but still comparable to the 34.1% reported from a nationwide American commercial insurer database in 2008 [44]. Moreover, the study conducted by Welch et al. focused on antihypertensive drug use in children and reported that among those with primary hypertension, the prescription rate was highest in the youngest age group (0 to <6 years, 36.5%) and lower in adolescents aged 12 to <18 years (35.0%). The most prescribed drug class in our study was calcium channel blockers, used in 16.8% of patients, followed by ACE inhibitors, used in 8.4% of patients. This differs from the previous data in which ACEi was the most commonly prescribed drug (14.8%), followed by calcium channel blockers (9%), in the previously mentioned study [44]. In both our study and the study conducted by Welch et al., beta antagonists were the third most used drug class [44]. However, while angiotensin receptor blockers (ARBs) were the least commonly prescribed medication in the U.S. data, loop diuretics were the least commonly prescribed drug class in our cohort [44]. Several factors may explain the differences in drugs prescribed in Welch et al.’s study compared to ours. Firstly, their study utilized incidence claims data, which encompassed prescriptions from a diverse range of healthcare professionals, including primary care doctors and highly specialized reference centers. Second, in the study conducted by Welch et al., the researchers used ICD-9 codes to identify children diagnosed with hypertension and required continuous insurance coverage for the full year of 2008 [44]. Finally, although Welch et al. did not report race or ethnicity data, the authors acknowledged that, in the United States, hypertensive children are disproportionately African American compared to other ethnicities [44]. It is also worth mentioning that the data in our study were collected during the first interview at our center, which may explain why calcium channel blockers were often chosen initially due to their relatively limited impact on diagnostic tests performed during hypertension evaluation.

Pathophysiologically, the current choice of drugs used to treat pediatric hypertension is closely linked to mechanisms regulating sodium and fluid homeostasis. Currently, according to the clinical guidelines, several types of drugs are recommended for the management of pediatric hypertension [1]. First-line drug treatments include angiotensin-converting enzyme (ACE) inhibitors, angiotensin receptor blockers (ARBs), calcium channel blockers (CCBs), and thiazide diuretics [45]. Diuretics were used in only 0.8% of the patients; these drugs promote direct renal sodium loss and are crucial in treating volume-dependent hypertension. However, their mechanism of action may induce electrolyte imbalances, and they are typically used in conjunction with the treatment of secondary hypertension [46]. It is worth noting that diuretics, by increasing diuresis, can lead to more frequent toilet visits during the day as well as during sleep at night, which can reduce quality of life for children. Additionally, fluid overload is typically absent in primary hypertension, highlighting the importance of carefully considering both the benefits and potential drawbacks of each medication when making treatment decisions [47]. The pharmacological choices for our study group suggest a preference for vascular-targeted therapies that target the RAAS and regulate vascular tone.

Vitamin D deficiency has been shown to activate the RAAS, resulting in higher renin levels, increased blood pressure, and decreased urinary sodium excretion in animal models [48]. In the human population, vitamin D deficiency may contribute to chronic overactivation of the renin–angiotensin system (RAS), explaining its connection to various cardiovascular and neuroinflammatory disorders [49]. McMullan et al. conducted a study in which the researchers demonstrated that higher vitamin D levels are associated with greater sodium excretion in hypertensive pediatric patients [50]. Similarly, our research found that 25-hydroxyvitamin D (25 OHD) levels correlated positively with urinary sodium excretion indices, suggesting that vitamin D sufficiency may promote renal sodium excretion. Our findings indicate that improving vitamin D status may be beneficial in treating pediatric hypertension in patients with RAAS overactivity or sodium retention.

In early childhood among children with primary hypertension, studies suggest that boys and girls have no differences in blood pressure [51]. However, after puberty, a clear male disparity can be observed [52,53]. Our findings are consistent with those data, confirming no sex predisposition to primary hypertension in children aged 12 and younger, with a clear male predominance after the age of 12. Children with primary hypertension are frequently overweight or obese [54]. A study by Halldorsson et al. concluded that boys that experience peak height velocity at an early age (8–13 years) had an increased risk of developing hypertension in adulthood [55]. In our study, children younger than 12 years had higher height Z-scores compared to those aged 12 years and older, while weight Z-scores did not differ between the groups. These findings suggest that taller younger children may be at a higher risk of developing primary hypertension. In our study, younger patients had higher total cholesterol and LDL cholesterol, which is consistent with observations in healthy populations showing a physiological peak in cholesterol levels among children aged 9 to 11 years [56]. A recent cross-sectional study by Baran et al. showed that healthy children aged 6–12 years had higher rates of lipid abnormalities than those aged 13 to 17 years [57]. In our study, ACE inhibitors were more frequently prescribed in younger children compared to their older peers. Similarly, in a recent study conducted by Ivanova et al., ACE inhibitors were the most commonly prescribed drug class to treat primary hypertension. Additionally, the authors showed that children aged less that 12 years were treated with ACEi more frequently than their older peers [58]. In our study, this may be attributed to the use of calcium channel blockers or alpha receptor blockers as the primary treatment for hypertension. In the absence of indications to modify therapy and with good treatment tolerance, patients typically remain on their initial regimen. This pattern suggests either more frequent indications for ACE inhibitors or ARB use among younger patients or a more severe course of hypertension in the younger group. However, further research is needed to confirm these observations.

### Strengths and Limitations

It is essential to acknowledge the strengths and limitations of the study. Firstly, to our knowledge, this study is one of the most extensive retrospective cross-sectional studies examining sodium excretion and blood pressure in hypertensive children, particularly in the central–eastern European population [12]. A significant additional advantage is the fact that the results were compared to a control group consisting of 59 healthy children without hypertension or any other chronic diseases. Both the study group and the control group underwent thorough blood pressure assessments, with each patient undergoing 24 h ambulatory blood pressure monitoring (ABPM). As a result, precise blood pressure parameters were recorded in a large cohort of children.

The most significant limitation of this study was the lack of information regarding the sodium intake of the participants in the study group due to the limited availability of precise data resulting from the study’s retrospective nature. We cannot rule out other more reliable test results if we had evaluated more than one urine sample and more than one daily urine collection. Another limitation, in addition to the study’s retrospective nature, is that the patients were recruited from a single medical center and represent a single racial group, lacking ethnic diversity. It should also be noted that a proportion of patients had been receiving antihypertensive therapy, most commonly as monotherapy, before undergoing urinary sodium collection or ambulatory blood pressure monitoring (ABPM) placement. Future studies should therefore be designed as prospective, multi-center investigations including ethnically diverse populations to validate and expand on our findings.

## 5. Conclusions

Children with primary hypertension excrete more sodium in their urine than healthy children. Since children with high sodium excretion had higher systolic blood pressure load and younger children had higher sodium excretion than older children, dietary intervention with a low sodium diet should be implemented more strongly in this group of patients. Children who are underweight for their age, young, and have a father with arterial hypertension might be at a higher risk of excessive urine excretion. Thus, these groups of children require more cautious urine sodium and dietary assessments. Due to the limited availability of data, additional longitudinal and interventional studies are needed to evaluate the effects of dietary interventions on blood pressure parameters in children with primary hypertension and to identify which children are most likely to benefit from such interventions.

## Figures and Tables

**Table 1:** Clinical characteristics of the study group.

Parameter	Study Group	Control Group	*p*
Number of patients (*n*)	386	59	
Age (years)	14.47 ± 3.00 (13.25–16.57)	15.29 ± 1.56 (13.76–16.56)	0.701
Boys/Girls	258/128 (66.8%/33.2%)	41/18 (69%/31%)	0.698
Height (cm)	166.9 ± 16.89 (161.0–178.0)	171.73 ± 10.52 (165.0–180.0)	0.316
Height Z-score	0.66 ± 1.12 (0.008–1.416)	0.57 ± 1.00 (−0.24–1.2)	0.452
Weight (kg)	71.56 ± 22.47 (60.0–84.0)	61.7 ± 11.50 (55.0–69.0)	<0.001
Weight Z-score	1.33 ± 1.10 (0.60–2.14)	0.43 ± 0.84 (0.01–1.03)	<0.001
BMI (kg)	25.17 ± 6.20 (21.24–28.32)	20.90 ± 3.40 (18.67–22.40)	<0.001
BMI Z-score	1.20 ± 1.10 (0.60–2.01)	0.17 ± 1.10 (−0.42–0.83)	<0.001
SBP (mmHg)	135.8 ± 12.61 (127.0–145.0)	118.81 ± 9.57 (113.0–126.0)	<0.001
SBP Z- score	1.96 ± 1.12 (1.35–2.71)	0.25 ± 0.81 (−0.34–0.85)	<0.001
DBP (mmHg)	81.42 ± 10.02 (75.0–87.0)	73.03 ± 6.26 (69.0–77.0)	<0.001
Father AH (yes/no)	38%/62%	29%/71%	0.193
Mother AH (yes/no)	20%/80%	8%/92%	0.032
Duration of gestation (weeks)	38.90 ± 2.00 (38.0–40.0)	38.70 ± 1.95 (38.0–40.0)	0.171
Birth weight (g)	3311.70 ± 583.31 (3040–3650)	3324.48 ± 582.97 (3160–3650)	0.352
Serum creatinine(mg/dL)	0.74 ± 0.18 (0.60–0.88)	0.77 ± 0.14 (0.67–0.86)	0.584
Serum UA (mg/dL)	5.56 1.50 ± (4.7–6.3)	4.97 ± 0.87 (4.5–5.7)	<0.001
Total cholesterol (mg/dL)	86.55 ± 24.85 (68.0–101.2)	83.57 ± 25.44 (67.0–95.0)	0.349
LDL cholesterol (mg/dL)	86.55 ± 24.85 (68.0–101.2)	83.57 ± 25.44 (67.0–95.0)	0.349
HDL cholesterol (mg/dL)	51.63 ± 12.98 (43.0–59.0)	57.62 ± 11.65 (49.0- 65.3)	<0.001
Serum sodium (mmol/L)	140.6 ± 2.71 (139.0–143.0)	139.3 ± 2.1 (138.0–140.0)	<0.001
Serum potassium (mmol/L)	4.36 ± 0.46 (4.1–4.6)	4.20 ± 0.68 (3.8–4.6)	0.019
Serum calcium (mg/dL)	9.92 ± 0.4 (9.70–10.2)	9.86 ± 0.34 (9.7–10.1)	0.356
LVM (g)	130.77 ± 44.44 (98.27–156.93)	122.22 ± 31.75 (103.10–144.10)	0.110
LVM Z-score	−0.42 ± 2.20 (−1.92- 0.85)	−2.34 ±1.02 (−2.60–−1.90)	<0.001
SBP 24 h (mm Hg)	127.23 ± 9.93 (120.0–134.0)	116.5 ± 7.3 (110.0–123.0)	<0.001
DBP 24 h (mm Hg)	70.59 ± 7.15 (66.0–75.0)	66.42 ± 5.44 (63.0–70.0)	<0.001
HR 24 h (bpm)	79.12 ± 10.53 (71.50- 87.0)	77.02 ± 9.33 (71.0–83.0)	0.350
PP 24 h (mm Hg)	55.46 ± 8.04 (51.0–62.0)	49.54 ± 6.36 (45.0–54.0)	<0.001
Dip sys (%)	11.21 ± 6.06 (7.45–15.20)	10.60 ± 5.22 (6.90–13.74)	0.463
Dip dia (%)	16.06 ± 8.08 (11.20–20.70)	17.78 ± 6.06 (13.51- 21.74)	0.023
24 h urinary sodium (mmol/kg/24 h)	2.23 ± 1.17 (1.45–2.87)	-	-
Na/creatinine (mmol/L/mg/dL)	1.53 ± 9.65 (0.64–1.33)	0.62 ± 0.34 (0.36–0.78)	<0.001
K/creatinine (mmol/L/mg/dL)	0.86 ± 6.49 (0.19–0.42)	0.18 ± 0.07 (0.13–0.20)	<0.001
Ca/creatinine (mg/mg)	0.12 ± 0.13 (0.05–0.13)	0.10 ± 0.06 (0.05–0.13)	0.624
UA/creatinine (mg/mg)	0.74 ± 5.07 (0.30–0.48)	0.34 ± 0.14 (0.25–0.40)	0.005
P/creatinine (mg/mg)	0.94 ± 6.18 (0.45–0.72)	0.81 ± 0.23 (0.65–0.90)	<0.001
ACR (mg/g)	27.22 ± 106.04 (3.61–11.32)	8.13 ± 8.00 (3.58–9.10)	0.263

AH—arterial hypertension, BMI—body mass index; SBP—systolic blood pressure; DBP—diastolic blood pressure; Z-score—Z-standard score; UA—urine acid; HDL—high-density lipoprotein; LDL—low-density lipoprotein; LVM—left ventricular mass; SBP 24 h—systolic blood pressure for 24 h, mmol/dL (millimoles per deciliter); DBP 24 h—diastolic blood pressure for 24 h; HR 24 h—heart rate for 24 h; MAP 24 h—mean arterial pressure for 24 h; PP 24 h—pulse pressure for 24 h; dip sys—systolic blood pressure drop; Dip dia—diastolic blood pressure drop; K—potassium; Ca—calcium; P—phosphorus; ACR—albumin-to-creatinine ratio.

**Table 2:** Correlations of sodium to creatinine urine excretion with various parameters of the study group.

Analyzed Parameter	Na/Creat (mmol/mg)			
	Study Group		Control Group	
	R	*p*	R	*p*
Age (years)	−0.261	<0.001	−0.251	0.056
Height (cm)	−0.233	<0.001	−0.239	0.068
Weight (kg)	−0.148	<0.001	−0.162	0.220
SBP Z-score	0.106	0.030	0.064	0.629
Duration of symptoms before diagnosis (months)	0.237	<0.001	-	-
UA (mg/dL) serum	−0.144	<0.001	−0.191	0.148
K (mmol/L) serum	0.171	<0.001	0.074	0.578
25 OHD (ng/mL)	0.157	<0.001	-	-
Z-score LVM	0.338	<0.001	−0.221	0.093
LVM (g)	−0.129	0.020	0.015	0.910
MAP 24 H	−0.268	<0.001	−0.121	0.362
K/Creat urine	0.629	<0.001	0.144	0.278
Ca/Creat urine	0.319	<0.001	0.437	<0.001
UA/creat urine	0.579	<0.001	0.672	0.033
ACR (mg/g)	0.122	0.010	−0.055	0.681

SBP (systolic blood pressure), Na (sodium), Creat (creatinine), K (potassium), ACEi (angiotensin-converting enzyme inhibitors), Z-score (standardized score), 25 OHD (25-hydroxycholecalciferol), UA (uric acid), LVM (left ventricular mass), MAP (mean arterial pressure), 24 H (24 h), UT (urine test).

**Table 3:** Correlations of 24 h urine sodium per kilogram of body weight with various parameters of the study group.

Analyzed Parameter	UT Na mmol/kg/24 h	
	Study Group	
	R	*p*
Weight	−0.352	<0.001
Weight Z-score	−0.299	<0.001
BMI	−0.354	<0.001
SBP	−0.126	0.030
DBP	−0.174	<0.001
DBP Z-score	−0.125	0.030
Age (years)	−0.162	<0.001
UA (mg/dL)	−0.203	<0.001
Cholesterol (mg/dL)	0.125	0.030
Cholesterol HDL (mg/dL)	0.177	<0.001
Triglycerides (mg/dL)	−0.125	0.030
25 OHD (pg/dL)	0.151	<0.001
LVM (g)	−0.149	0.040
K/Creat urine	0.343	<0.001
UA/Creat urine	0.175	0.020
P/Creat urine	0.210	<0.001

4 H UT Na/kg bw (24 h urine sodium per kilogram of body weight), BMI (body mass index), SBP (systolic blood pressure), DBP (diastolic blood pressure), Father AH (arterial hypertension of father), Z-score (standardized score), AD (antihypertensive drugs), Cabloker (calcium channel blocker), UA (uric acid), 25 OHD (25-hydroxycholecalciferol), K (potassium), P (phosphorus), mg/g (milligrams per gram), UT (urine test).

**Table 4:** Comparison of selected parameters between subgroups with normal and excessive sodium excretion.

Parameter	Normal Sodium Urine Excretion *n* = 347	Excessive Sodium Excretion *n* = 22	*p*
Age (years)	14.94 ± 2.28 (13.70–16.63)	12.05 ± 3.28 (13.25–16.57)	<0.001
Height (cm)	169.43 ± 12.71 (163.0–178.5)	154.40 ± 19.43 (137.5–170.0)	<0.001
Height Z-score (cm)	0.67 ± 1.16 (0.008–1.416)	0.73 ± 0.86 (0.11–1.32)	0.788
Weight Z-score (kg)	1.34 ± 1.06	1.02 ± 1.09	0.195
	(0.602–2.141)	(0.14–1.77)	
BMI	25.46 ± 5.95 (21.51–28.53)	22.04 ± 6.07 (17.98–24.49)	0.004
BMI Z-score	1.22 ± 1.07 (0.57–2.01)	0.81 ± 1.18 (−0.294–1.68)	0.111
SBP	136.86 ± 12.46 (128.0–145.0)	132.21 ± 11.35 (120.0–139.0)	0.114
SBP Z-score	2.04 ± 1.10 (1.36–2.83)	1.97 ± 1.12 (1.34–2.5)	0.801
DBP	81.92 ± 10.02 (76.0–88.0)	79.37 ± 11.41 (72.0–86.0)	0.286
DBP Z-score	2.31 ± 1.38 (1.47–3.1)	2.14 ± 1.51 (1.342–2.95)	0.602
Duration of symptoms (months) before diagnosis	15.92 ± 22.19 (3.0–19.0)	14.39 ± 24.04 (3.0–8.0)	0.778
Hbd	38.98 ± 1.69 (38.0–40.0)	40.11 ± 1.27 (40.0–40.0)	0.053
Birth weight (g)	3319.31 ± 617.29 (3000.0–3650.0)	3414.44 ± 343.11 (3280.0–3650.0)	0.649
UA (mg/dL)	5.62 ± 1.55 (22.3–51.4)	5.09 ± 1.42 (19.70–54.90)	0.165
Cholesterol (mg/dL)	155.05 ± 28.87 (134.0–175.0)	161.79 ± 22.39 (151.0–181.0)	0.320
Cholesterol LDL (mg/dL)	85.02 ± 24.76 (67.4–99.2)	91.06 ± 21.43 (75.40–109.0)	0.314
Cholesterol HDL (mg/dL)	50.63 ± 11.66 (43.0–58.0)	57.90 ± 15.43 (41.0–72.0)	0.046
Triglyceride (mg/dL)	96.49 ± 44.67 (64.0–119.0)	69.16 ± 32.95 (47.0–86.0)	0.001
Potassium concentration plasma (mmol/L)	4.37 ± 0.36 (59.0–127.0)	4.57 ± 0.40 (4.30–4.70)	0.041
Sodium concentration plasma (mmol/L)	140.89 ± 2.82 (139.0–143.0)	140.42 ± 2.17 (139.0–142.0)	0.480
LVM (g)	130.68 ± 42.51 (120.0–134.0)	108.30 ± 42.10 (82.5–133.7)	0.091
Z-score LVM	0.28 ± 2.00 (−1.20–1.25)	0.12 ± 2.29 (−1.00–0.95)	0.800
SBP 24 h	127.35 ± 10.02 (13.25–16.57)	127.37 ± 8.76 (120.0–133.0)	1.000
DBP 24 h	70.25 ± 7.12 (65.0–75.0)	71.79 ± 7.47 (67.0–76.0)	0.364
HR 24 h	78.31 ± 10.38 (71.0–87.0)	82.79 ± 10.65 (72.0–90.0)	0.070
SBPL/24 h (%)	38.51 ± 26.21 (17.0–55.0)	51.95 ± 25.82 (35.0–71.0)	0.031
DBPL/24 h (%)	19.92 ± 19.51 (6.0–28.0)	26.42 ± 25.47 (9.0–37.0)	0.170
Dip sys	10.97 ± 5.95 (7.4–14.9)	13.72 ± 6.30 (7.5–18.7)	0.054
Dip dia	15.80 ± 8.27 (11.2– 20.4)	18.59 ± 8.93 (11.81–25.6)	0.157
P/Creat	1.03 ± 7.24 (0.42–0.64)	0.71 ± 0.30 (0.51–0.91)	0.866
ACR (mg/g)	31.73 ± 120.81 (3.63–11.87)	18.32 ± 27.24 (19.4–27.24)	0.630

BMI—body mass index; Z-score—Z-standard score; SBP—systolic blood pressure; DBP—diastolic blood pressure; UA—urine acid; HDL—high-density lipoprotein; LDL—low-density lipoprotein; LVM—left ventricular mass; SBP 24 h—systolic blood pressure for 24 h; DBP 24 h—diastolic blood pressure for 24 h; HR 24 h—heart rate for 24 h; SBPL/24 h(%)—systolic blood pressure load over 24 h %; DBPL/24 h (%)—diastolic blood pressure load over 24 h %; dip sys—systolic blood pressure drop, Dip dia—diastolic blood pressure drop; P—phosphorus; ACR—albumin-to-creatinine ratio.

**Table 5:** Comparison of selected parameters between subgroups of children younger and older than 12 years of age.

Parameter	<12 Year Old	>12 Year Old	*p*
Age (years)	10.19 ± 1.37 (9.43–11.34)	15.58 ± 1.55 (14.53–16.79)	<0.001
Sex (Boys/Girls)	54%/46%	69%/31%	0.045
Height (cm)	147.5 ± 12.6 (9.4–11.3)	172.5 ± 9.7 (166.5–180.0)	<0.001
Height Z-score	1.28 ± 1.26 (0.55–2.11)	0.56 ± 1.07 (−0.08–1.31)	<0.001
Weight (kg)	51.2 ± 21.5 (33.0–66.1)	76.99 ± 18.20 (63.7–86.4)	<0.001
Weight Z-score	1.38 ± 1.19 (0.55–2.38)	1.34 ± 1.05 (0.62–2.08)	0.600
BMI	22.83 ± 6.69 (17.69–27.74)	25.86 ± 5.92 (21.82–28.45)	0.003
BMI Z-score	1.09 ± 1.39 (0.41–2.04)	1.24 ± 1.03 (0.57–1.96)	0.849
SBP	127.04 ± 10.40 (118.50–133.00)	137.99 ± 11.71 (130.00–146.00)	<0.001
SBP Z-score	1.80 ± 1.41 (1.16–2.48)	2.03 ± 1.04 (1.38–2.73)	0.154
DBP	78.83 ± 8.09 (74.00–83.00)	82.21 ± 9.96 (76.00–88.00)	0.007
DBP Z-score	2.17 ± 1.19 (1.37–2.90)	2.30 ± 1.36 (1.49–3.10)	0.320
Duration of symptoms before diagnosis (months)	12.95 ± 16.44 (3 −12)	15.19 ± 21.86 (2–18)	0.909
Father AH (yes/no)	40.5%	37%	0.666
Mother AH (yes/no)	17%	20%	0.571
Hbd (weeks)	38.7 ± 2.2 (37.0–40.0)	38.9 ± 1.9 (38.0–40.0)	0.652
Birth weight (g)	3259 ± 467 (2950–3570)	3313 ± 611 (3040–3650)	0.361
Antihypertensive drugs	26%	27%	0.916
ACEi	17%	7%	0.032
UA (mg/dL)	4.97 ± 1.56 (4.10–5.80)	5.72 ± 1.46 (4.80–6.50)	<0.001
Total cholesterol (mg/dL)	167.9 ± 27.3 (148.0–184.0)	155.1 ± 29.5 (134.0–176.0)	0.005
Cholesterol LDL (mg/dL)	94.4 ±26.6 (79.2–110.0)	85.1 ± 24.6 (66.4–99.0)	0.030
Cholesterol HDL (mg/dL)	53.3 ±12.0 (45.0–59.0)	51.0 ± 12.6 (42.0–58.0)	0.235
Triglyceride (mg/dL)	101.7 ± 44.7 (64.0–137.0)	94.7 ± 44.8 (62.0–118.0)	0.241
K blood (mmol/L)	4.55 ± 0.51 (4.30–4.75)	4.31 ± 0.42 (4.10–4.50)	0.004
Na blood (mg/dL)	140.1 ± 2.9 (138.0–142.0)	140.7 ± 2.7 (139.0–143.0)	0.301
25-OH-D (pg/mL)	25.6 ± 12.6 (18.5–30.0)	24.1 ± 12.4 (16.4–29.2)	0.198
Z-score LVM	−0.59 ± 2.31 (−0.17–0.24)	−0.36 ± 2.19 (−1.84 ± 0.87)	0.437
LVM (g)	89.18 ± 35.38 (63.21–110.76)	139.07 ± 40.55 (108.79–158.82)	<0.001
SBP 24 h	122.6 ± 7.9 (117–127)	128.3 ± 9.8 (122–135)	<0.001
DBP 24 h	70.1 ± 5.2 (67–73)	70.6 ± 7.3 (65–75)	0.891
HR 24 h	87.3 ± 8.1 (83–93)	77.3 ± 9.9 (70–83)	<0.001
MAP 24 h	86.9 ± 7.1 (81–92)	89.7 ± 8.6 (84–96)	0.038
MAP 24 h Z-score	0.94 ± 1.10 (0.28–1.52)	0.79 ± 1.46 (−0.14–1.62)	0.318
PP 24 h	52.5 ± 6.1 (48.0–57.0)	57.5 ± 8.0 (52.0–63.0)	<0.001
SBPL/24 h (%)	43.4 ± 26.4 (19.0–67.0)	38.8 ± 26.4 (17.0–56.0)	0.299
DBPL/24 h (%)	22.0 ± 19.6 (8.0–29.0)	20.1 ± 20.1 (6.0–27.4)	0.300
Dip sys	10.6 ± 4.5 (7.6–13.3)	11.1 ± 6.3 (7.2–15.2)	0.372
Dip dia	17.4 ± 6.1 (12.8–23.0)	15.6 ± 8.3 (10.9–20.4)	0.137
Na/Creat (mmol/mg)	1.59 ± 0.81 (1.01–2.15)	0.92 ± 0.45 (0.60–1.20)	<0.001
K/Creat (mmol/mg)	0.48 ± 0.26 (0.26–0.68)	0.31 ± 0.18 (0.18–0.38)	0.005
Ca/Creat (mg/mg)	0.11 ± 0.10 (0.04–0.14)	0.10 ± 0.06 (0.60–0.13)	0.709
UA/Creat (mg/mg)	0.57 ± 0.19 (0.44–0.70)	0.38 ± 0.31 (0.29–0.41)	<0.001
P/Creat	0.78 ± 0.38 (0.61–0.94)	0.54 ± 0.19 (0.44–0.65)	<0.001
ACR (mg/g)	6.7 * ± 20.0 (5.1–22.5)	5.3 * ± 38.5 (3.3–10.0)	0.006
Na mmol/kg/24 h	2.95 ± 1.48 (1.79–3.93)	2.04 ± 0.97 (1.40–2.65)	<0.001

*—median; BMI—body mass index; SBP—systolic blood pressure; DBP—diastolic blood pressure; Z- score—Z-standard score; AH—arterial hypertension; Hbd—weeks of pregnancy; ACEi—angiotensin-converting-enzyme inhibitors; UA—uric acid; HDL—high-density lipoprotein; LDL—low-density lipoprotein; LVM—left ventricular mass; SBP 24 h—systolic blood pressure for 24 h; DBP 24 h—diastolic blood pressure for 24 h; HR 24 h—heart rate for 24 h; MAP 24 h—mean arterial pressure for 24 h; PP 24 h—Pulse Pressure for 24 h; Dip sys— nighttime systolic blood pressure dip; Dip dia—nighttime diastolic blood pressure dip; K—potassium; Ca—calcium; P—phosphorus; ACR—albumin-to-creatinine ratio.

## Data Availability

Raw data are accessible on request from the authors.
